# A Comparison of Screening Methods for Sleep Disorders in Australian Community Pharmacies: A Randomized Controlled Trial

**DOI:** 10.1371/journal.pone.0101003

**Published:** 2014-06-30

**Authors:** Joanne M. Fuller, Keith K. Wong, Ronald Grunstein, Ines Krass, Jayshree Patel, Bandana Saini

**Affiliations:** 1 Faculty of Pharmacy, The University of Sydney, Sydney, NSW, Australia; 2 The Centre for Integrated Research and Understanding of Sleep (CIRUS), The University of Sydney, Sydney, NSW, Australia; 3 Royal Prince Alfred Hospital, Sydney, NSW, Australia; 4 The Woolcock Institute of Medical Research, The University of Sydney, Sydney, NSW, Australia; Iran University of Medical Sciences, Islamic Republic of Iran

## Abstract

**Background:**

Community pharmacies may potentially assist in screening for chronic conditions such as sleep disorders, which remain both under-diagnosed and untreated. We aimed to compare a subjective risk-assessment-only questionnaire (RAO) for common sleep disorder screening against the same risk-assessment questionnaire *plus* a nasal flow monitor as an objective marker of possible underlying obstructive sleep apnea (OSA) (RA+) in a community pharmacy setting. The primary outcome was the number of participants identified in RAO or RA+ group who were likely to have and consequently be diagnosed with OSA. Further outcomes included the number of participants identified as being at risk for, referred for, taking-up referral for, and then diagnosed with OSA, insomnia, and/or restless legs syndrome (RLS) in either group.

**Methods:**

In a cluster-randomized trial, participants were recruited through 23 community pharmacies. Using validated instruments, 325 (RAO = 152, RA+ = 173) participants were screened for OSA, insomnia, and RLS.

**Findings:**

218 (67%) participants were at risk of OSA, insomnia or RLS and these participants were referred to their primary physician. The proportion of screened participants identified as being at risk of OSA was significantly higher in the RA+ group (36% in RAO vs. 66% in RA+, OR 3.4, 95% CI (1.8–6.5), p<0.001). A 12-month follow-up was completed in 125 RAO and 155 RA+ participants. Actual referral uptake was 34% RAO, 26% RA+, OR 4.4, 95% CI (1.4–19.2), p = 0.31. The OSA diagnosis rate was higher in the RA+ arm (p = 0.01). To yield a single additional confirmed OSA diagnosis, 16 people would need to be screened using the RA+ protocol.

**Conclusions:**

These results demonstrate that utilising either screening method is feasible in identifying individuals in the community pharmacy setting who are likely to have OSA, insomnia and/or RLS. Secondly, adding an objective marker of OSA to a questionnaire-based prediction tool resulted in more confirmed OSA diagnoses.

**Trial Registration:**

: ACTR.org.au ACTRN12608000628347

## Introduction

Sleep disorders remain both under-diagnosed and untreated despite being highly prevalent and imposing substantial burden in the developed world [1]–[2]. In Australia, published data indicates that sleep disorders affect 1.5 million individuals (8.9%) and result in a total estimated cost of $AUD36.4 billion [2]. Common sleep disorders affecting Australians comprise obstructive sleep apnea (OSA) (4.7%), primary insomnia (3%), and restless legs syndrome (RLS) (1.2%) [2]. Furthermore, sleep disorders are commonly associated with other major medical problems [3]–[4] and therefore present more frequently in the primary health care population. Sleep disturbances, such as those caused by obstructive sleep apnea (OSA) and insomnia, are associated with increased likelihood of cardiovascular disease, diminished quality of life, and increased all-cause mortality risk, and are now considered one of the top 10 potentially modifiable cardiovascular disease risk factors [1], [5]–[8]. Early recognition and treatment of common sleep disorders could therefore help minimize significant health, social and fiscal consequences [9]. With heightening public awareness of sleep disorders, concern has been raised about inadequate diagnostic and therapeutic capacity in the community [10]. This supply-demand mismatch drives the need to explore alternative approaches to facilitate earlier diagnosis including alternatives to the standard diagnostic tests [1].

Research in alternative diagnostic pathways or screening protocols for common sleep disorders has focused on specialist sleep clinics, or more recently, in primary care [10]. However, many people with sleep disorders do not seek help from a physician, but choose to self-medicate [11]. Hence, other settings accessed by those who do not engage with the medical system, need to be considered. Community pharmacists represent a primary healthcare resource with unrealized capacity to contribute to health promotion and screening through collaboration with physicians. In the United Kingdom, community pharmacy is recognized as a “valuable and trusted public health resource” with several preventative and public health programs delivered through pharmacies [12]. Furthermore, pharmacy based screening programs for various chronic diseases have been shown to be effective [13]–[15]. However, there are very few published studies on pharmacy-based screening for sleep disorders. A Swiss questionnaire-based sleep disorder screening program identified 26% of the screened population as being at risk of having a sleep disorder [16]. Those identified at risk in the study were consequently referred to a physician by trained community pharmacists [16]. Another questionnaire-based screening tool (Pharmacy Tool for Assessment of Sleep Health (PTASH)) was recently piloted in five Australian pharmacies and shown to be feasible in screening for the most common sleep disorders [17]. This questionnaire targeted three prevalent disorders (restless legs syndrome (RLS), OSA, and insomnia), and utilized validated instruments i.e. the Epworth Sleepiness Scale (ESS) [18], Insomnia Severity Index (ISI) [19], Multivariable Apnea Prediction Index (MAPI) [20], and the NIH Restless legs syndrome workshop diagnostic criteria [21]. Using this composite questionnaire, the study assessed sleep disorder risk in a small sample of 85 patients recruited through community pharmacies in Sydney, Australia. Results indicated that 33.3%, 21.4%, and 27.4% of those screened were likely to have insomnia, OSA, and RLS respectively [17].

Neither of these pharmacy-based screening programs explored outcomes in terms of the proportion of screened population that was subsequently diagnosed by medical practitioners. Moreover, both previous studies only used a subjective assessment of sleep disorder likelihood. Simplified measures for OSA risk screening such as portable nasal flow monitors may offer community pharmacists an accurate but low cost method of objective risk assessment [22]–[23]. Objective measures for sleep disorders have not been studied in community pharmacy to our knowledge, however other objective measures have been employed in pharmacy based screening studies for cardiovascular risk and osteoporosis conducted in pharmacy populations [15], [24]. Apart from added accuracy [25], an objective measure may be more likely to influence patients and health care professionals to act upon the results of such a test [14]. However to date, simplified and relatively inexpensive (when compared to PSG) objective measures are only available for OSA assessment. Insomnia and RLS are typically assessed in primary care with subjective measures only.

In order to test the utility of adding an objective test to community pharmacy screening for sleep disorders, we compared two screening strategies: a subjective risk assessment only (RAO) protocol (using the PTASH questionnaire cited above [17]) versus a risk assessment plus objective marker of possible underlying OSA (RA+). (For the purposes of this study, the term ‘at risk’ pertains to those participants who are *likely to have* any of the sleep disorders being screened for.) Since the objective marker in the RA+ arm pertained to OSA only, we hypothesized that of the total patients screened in both groups, the RA+ group would have a higher total number of patients identified as being at risk of OSA and also diagnosed with OSA. This was the primary outcome. In addition, we expected that the RA+ group would report higher rates of being referred and taking up their referral. Other outcomes of interest included the number of patients identified as being at risk for OSA, insomnia and/or RLS, as well as the number of patients who were subsequently diagnosed with any of the three sleep disorders.

## Methods and Materials

### Ethics statement

All procedures were approved by the Human Research Ethics Committee at the University of Sydney (reference: 06-2008/10765, see additional supporting information). Written informed consent was obtained from all participants. The trial was listed on the Australian and New Zealand Clinical Trials Register (ACTRN12608000628347). See https://www.anzctr.org.au/Trial/Registration/TrialReview.aspx?ACTRN=12608000628347

### Study design

The study was a cluster randomized controlled trial, with individual pharmacists randomized to deliver one of two interventions to their patients. The supporting CONSORT checklist is available as supporting information; see Checklist S1. The study was not set-up as a diagnosis accuracy study as diagnosis does not fall within the pharmacy practitioner role.

### Pharmacist recruitment

A purposively selected sample of community pharmacies supplying sleep apnea devices was constructed from distributor lists of continuous positive airway pressure (CPAP) manufacturers; this comprised the total sampling frame. The pharmacies were categorised as either urban or non-urban, based on the proximity (i.e. being within a 100 km radius) of each pharmacy location to an urban centre (defined as a centre with a population of greater than 200,000 inhabitants). After recruiting all pharmacists (pharmacies) who were willing to participate in the project, a randomisation sequence was generated using a block size of 2 (urban/non-urban pharmacies), list length of 24, and an allocation ratio of 1:1 into either RAO or RA+ groups. The participating pharmacies were allocated to either the RAO or the RA+ group based on the sequence. Over a two-day workshop run by the research team, all recruited pharmacists were trained in basic sleep disorder issues, identifying patients with risk factors for sleep disorders, providing those at risk with appropriate referral/counselling, and in using the research protocol. The RAO and RA+ groups were trained separately in research protocol with the RA+ group receiving additional training on procedures for the portable monitor testing. This was in line with the cluster randomisation to preserve treatment fidelity and to ensure a consistent intervention would be applied to all recruited patients without any cross contamination.

### Patient recruitment

Patients were recruited by pharmacists over a four-month period, and were included if they were aged 18 years or above, not undergoing treatment for any sleep disorder, physically able to complete the screening in the pharmacist's opinion, fluent in English, and able to provide written consent. Patients were recruited either when requesting non-prescription products for sleep or information relating to sleep health, or when identified by the trained pharmacists as having possible risk factors for a sleep disorder, or in response to promotional materials.

### Screening protocol

**Figure 1:**
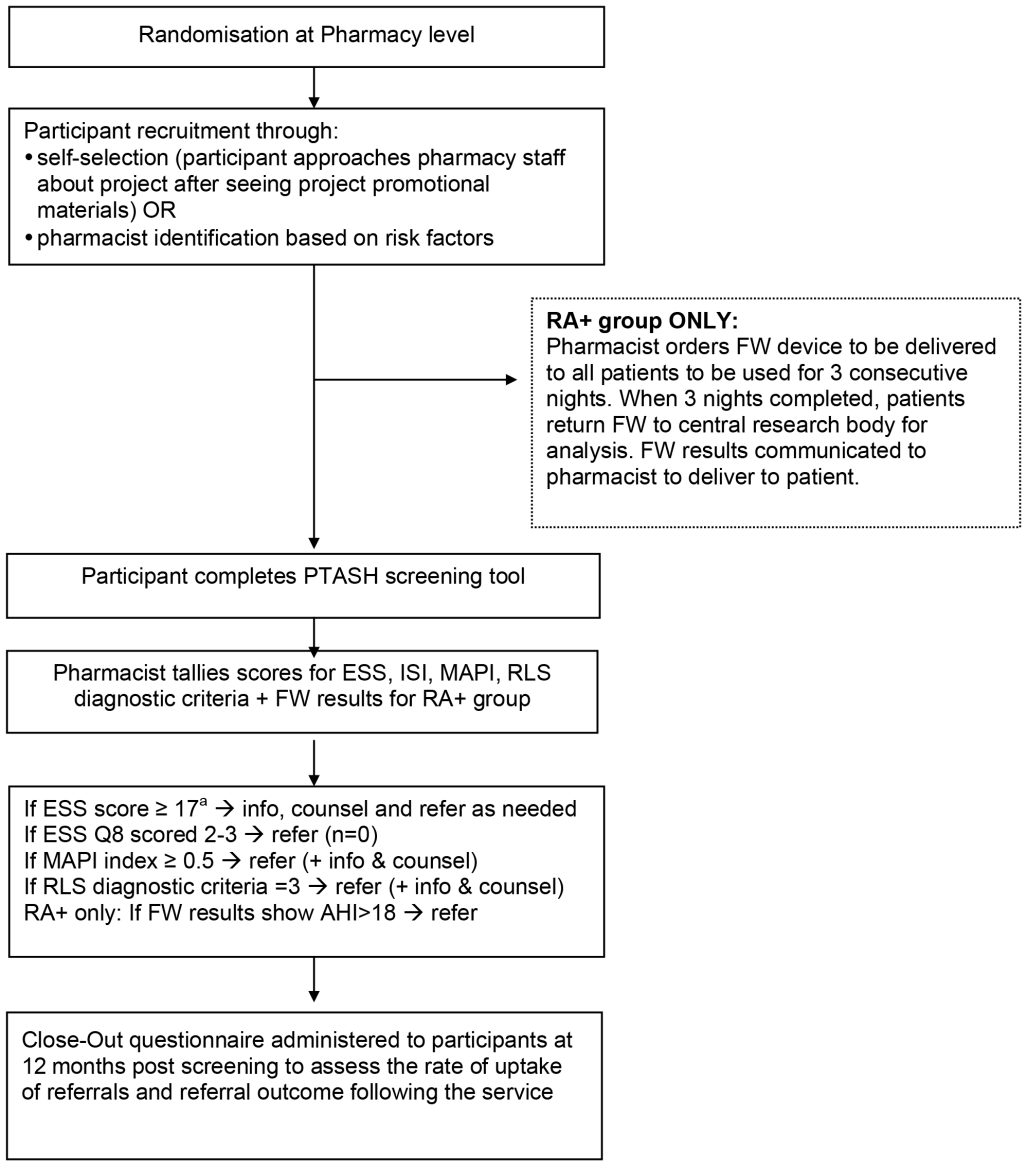
Project protocol for recruitment, screening, and referral and/or counselling of participants according to risk scores. Risk scores were obtained from the PTASH screening questionnaire and the FlowWizard (as an objective marker of SDB). ^a^ Clinical cut-off [22]–[23] RA+  =  Risk Assessment Plus, FW =  FlowWizard (nasal flow monitor) ESS =  Epworth Sleepiness Scale ISI =  Insomnia Severity Index MAPI =  Multivariate Sleep Apnea Prediction Index RLS =  Restless Legs Syndrome Info =  provision of sleep disorder specific written information AHI =  Apnea Hypopnoea Index PTASH =  Pharmacy Tool for Assessment of Sleep Health SDB =  sleep disordered breathing.

The development and pilot testing of the PTASH screening tool has been described elsewhere [17]. The PTASH questionnaire screens participants for being at risk of OSA, insomnia and/or RLS. The PTASH questionnaire also collects lifestyle, medical status, medication use and demographic information as these factors have been shown to influence sleep health. All participants were screened as described below, with the full study protocol available as Protocol S1, and illustrated in Figure 1.

In the RAO group, following application of the screening protocol (see Protocol S1), a ‘risk score’ for each of the three sleep disorders targeted by the PTASH screening tool was calculated, and the appropriate intervention (written information provision, in-depth counselling and physician referral) was implemented by the pharmacist. A participant that scored 17 or more on the ESS would be counselled by the pharmacist and/or referred to their physician as needed. All participants that scored 1 to 3 on question 8 of the ESS were referred to their physician. A participant that scored 0.5 or more on the MAPI survey was referred to their physician and counselled as needed by the pharmacist. A participant that scored 3 on the NIH Restless legs syndrome workshop diagnostic criteria was referred to their physician and counselled as needed by the pharmacist. In the RA+ group, patients were additionally asked to use a type 4 portable nasal flow monitor (FlowWizard, DiagnoseIT, Sydney, Australia) at home for three consecutive nights to provide an objective measure of OSA risk [22]–[23]. The flow monitor has been validated as a home test for OSA, in patients seen in the primary care setting. An Apnea-Hypopnea Index (AHI) from the portable monitor of greater than 18 (for the RA+ group) or the risk scores calculated from the PTASH screening tool, were used by the pharmacist to determine need for referral to a physician.

A letter, based on a template including questionnaire scores and their interpretation, was given to all referred patients by the pharmacist, with a recommendation that it be taken to their primary care physician for a medical evaluation.

### Sample size

The sample size was based on the study by Hersberger et. al. where 26% of the study population screened in community pharmacy settings in Switzerland were ‘at risk’ for a sleep disorder [16]. We assumed that in the RA+ group, the proportion of patients identified and referred would be equivalent to the Swiss study i.e. 26%, and that in the RAO group, with no objective measure, the proportion of people identified with a sleep disorder would be 50% lower, i.e. 13%. To detect a 13% point difference in referral rate between the RAO, and RA+ screening groups, with 80% power and significance level of 0.05, 114 patients were required in each group. Allowing for a 20% withdrawal rate, 143 patients per group were estimated to be required. We then applied a design effect correction. Given our experience of negligible design effect in other clustered trials within Australian community pharmacies, 0.004 was arbitrarily used as an estimate of the Intra Cluster Correlation coefficient (ICC) for our cluster based protocol. This value of 0.004 was also the lower end of ICC values observed in many clinical trials [26]. The correction factor was then calculated using a cluster size of 10 (as all pharmacies were requested to screen at least 10 patients), and the arbitrarily estimated intra-cluster correlation coefficient of 0.004 (1+(10−1)*0.004 = 1.036) [26], resulting in a desired size of 148 patients per group.

### Data analyses

Statistical analyses were conducted using SPSS TM version 18.0 and R (www.r-project.org) [27]. For continuous outcomes, linear mixed models analyses were conducted with SPSS TM. Fixed effects were entered as the group (RAO or RA+), and the model included a random intercept, grouped by the cluster variable (each individual pharmacy). The difference between the RAO and RA+, adjusted for the effect of clustering, was thus derived from the model, with 95% confidence limits and p-values. For binary outcome variables, raw proportions are quoted for each group (ignoring clustering), and then the differences between the two interventions adjusted for clustering are analysed with generalised linear mixed models with a logit link, using the lme4 function in R, and presented as odds ratios for the RA+ group relative to RAO, 95% confidence limits and a p value. Statistical significance was determined by a p<0.05.

### Diagnostic characteristics of the screening tool: Polysomnography (PSG sub-study)

Furthermore, a subset of 20 randomly selected RA+ patients underwent fully attended overnight PSG to assess diagnostic characteristics of the questionnaire screening tool and portable monitor in predicting OSA risk in the community pharmacy setting. The PSG sub-study was intended as an internal check, to evaluate if the screening procedure resulted in a large number of false positives. The number of 20 patients to be studied was determined by the limited funds and personnel resources available. These 20 patients were selected from a sampling frame of the overall study patients who were located in the metropolitan Sydney area, so that all could attend PSG testing in a central accredited facility. This sub-study was not sufficiently powered to make comparisons in performance between the two screening methods (questionnaire vs. portable monitoring). A sample size of 60–100 would have been required, for example, to detect differences in sensitivity or specificity of 0.2, with 80% power and significance level of 0.05, assuming the sensitivity or specificity of the better test was between 0.6–0.9.

## Results

### Pharmacy recruitment and randomization

Twenty-three of 35 pharmacies approached consented to participate; 12 were allocated to the ‘risk assessment only’ (RAO) group and 11 into the ‘risk assessment plus’ overnight nasal flow-monitoring (RA+) group. Two pharmacies from the RA+ group withdrew, and one from the RAO group withdrew (all metropolitan) due to staffing problems. Sixty percent of the completing pharmacies were non-metropolitan (RAO = 7, RA+ = 5). As an indication of the activity level of the participating pharmacies, dispensing levels were at par with national averages [28]. Although exact comparison of pharmacy by group type were not conducted, most pharmacies in Australia that supply CPAP are usually larger in size and staff levels [29].

### Participant characteristics & follow-up

**Figure 2:**
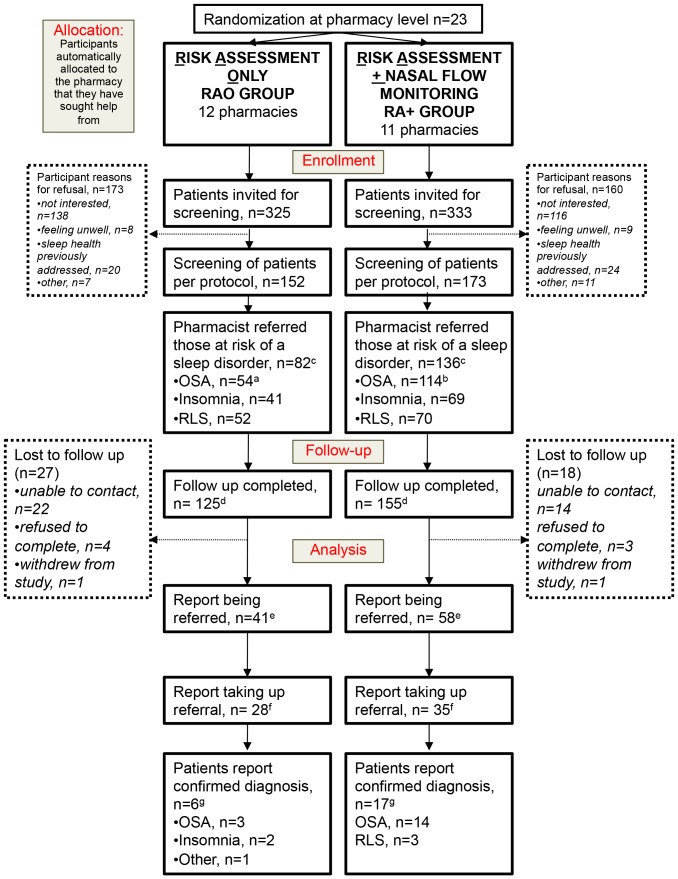
CONSORT diagram showing participant flow throughout the study. ^a^ Participants may be classified as at risk of more than one condition. ^b^ Comprises n = 76 identified by questionnaire and additional 38 identified by flow monitor AHI≥18 alone in the RA+ group. Comparison between groups: ^c^ number at risk of a sleep disorder as identified by pharmacist as a proportion of number screened, OR 3.2, 95% CI 1.7–6.3, p = 0.0009. ^d^ follow-up completed as proportion of number screened OR 1.6, 95% CI 0.54–4.5, p = 0.39. ^e^ Participants who report being referred as proportion of number referred, in those completing follow-up, OR 1.3, 95% CI 0.69–2.7, p = 0.40. ^f^ Participants who report taking up referral (i.e. actually seeing their medical practitioner) as proportion of those referred and completing follow-up, OR 0.65, 95% CI 0.26–1.6, p = 0.31. ^g^ Participants diagnosed as proportion of numbers screened, OR 2.7, 95% CI 1.1–7.5, p = 0.03.

The CONSORT ‘Participant Flow Diagram’ is shown in Figure 2. Twelve pharmacies in the RAO group recruited 152 participants, while 11 pharmacies in the RA+ group recruited 173 patients. Patient recruitment rate in the RAO (46.7%, 152/325) and the RA+ (51.9%, 173/333) were quite similar. The majority of participants were identified by pharmacists based on risk factors (n = 201) with a further 49 participants self-selecting (that is, approaching pharmacy staff about the project after seeing project promotional materials). The method of participant identification was not recorded by 23% of pharmacists. Two hundred and eighty patients overall (86%) completed follow-up at 12 months to assess the rate of uptake of referrals and referral outcome. Table 1 shows general demographic and sleep characteristics for the screened population. We tested for any between group differences that may have existed at baseline and found there were no significant differences between the two groups in age, gender, employment, percentage working shifts, diagnosis of hypertension, ethnicity, alcohol or caffeine or tobacco consumption. There were no significant differences in questionnaire-assessed risk of insomnia, OSA or RLS. Mean ISI score was not significantly different between the two intervention groups, nor was the proportion with a score of 15 or more which indicates higher risk of insomnia, (RA+ group 40.5% (69/173) vs. RAO group 27.3% (41/152), OR 2.2, 95% CI 0.89–6.0, p = 0.08).

**Table 1:** General Demographics and Sleep Characteristics in the two groups.

Characteristic	RAO group n = 152	RA+ group n = 173	Cluster-adjusted difference between groups, Mean or Odds Ratio (95% CI value)	p value (between group comparisons)
Mean Age ± SD	55.5±16.7	54.4 ±15.1	1.21(−5.4 −7.8)	0.71
% Male	46.7	47.9	0.05	0.82
Mean BMI±SD	29.7±5.4	29.9±7.0	0.06(−2.8 −2.9)	0.96
Sleepiness (ESS) % ESS>10	18.1	28.0	4.4	0.05
Mean ESS±SD *ESS score range 0–24*	5.6±4.2	7.0±4.8	−1.4(−2.8 −0.09)	*0.06*
Insomnia (ISI) % ISI≥15	27.3	40.5	*6.2*	*0.01*
Mean ISI Score ± SD *ISI Score range 0*–*28*	10.7±6.9	12.1±6.9	−2.0 (−5.3−1.2)	0.21
Sleep apnea risk (MAPI) % MAPI ≥0.5	37.0	44.1	1.7	0.20
Mean MAPI ± SD *MAPI probability range 0*–*1*	0.41±0.3	0.43±0.3	−0.02(−0.10−0.06)	0.65
At risk of RLS (RLS minimum question set) % responding ‘Yes’ to questions 1–3	42.6	46.6	0.45	0.54
Mean RLS Score ± SD, *RLS Score Range 0*–*3*	1.5±1.4	1.6±1.4	−0.30(−0.97 −0.37)	0.36
% Sleeping alone	37.5	45.1	1.91	0.18
% Undertaking shift work	11.3	9.3	1.5	0.46
% Consuming caffeine after 1500 hours	67.8	64.0	0.49	0.54
% Consuming alcohol after 2000 hours	50	54.2	0.46	0.53
Self reported average hours of sleep	6.46±1.7	6.36±1.7	0.19(−0.40–0.80)	0.51

### Screening outcomes for sleep disorders risk

All patients identified as at risk were provided with referrals to their primary care physician for further evaluation. The RA+ group (79%, 136/173) had a significantly higher rate of being identified as being ‘at risk’ of having any sleep disorder and being referred as per protocol compared to the RAO group (54%, 82/152, OR 3.22, 95% CI 1.7–6.3 p<0.0001). The proportion of screened patients identified as being at risk of having OSA was also significantly higher in the RA+ group (36% (54/152) in RAO vs. 66% (114/173) in RA+, OR 3.4, 95% CI 1.8–6.5 p<0.0001). While OSA risk was determined by questionnaire alone in the RAO group, in the RA+ group, patients were classified as at risk of OSA by either questionnaire or flow monitor, and consequently an additional 38 patients were classified as at risk of OSA (see figure 2) through testing with the flow monitor over what the questionnaire identified. The number of patients identified at risk for insomnia or RLS are shown in Figure 2. The mean time taken to complete the screening was longer in the RAO arm (12.7±9.0 minutes) compared to the RA+ arm (10.6±4.5 minutes). Using a linear mixed model approach, the estimated difference between the groups adjusting for the effect of clustering was 2.14 minutes (95% CI −0.44–4.7), and was not significant (p = 0.099). The overall mean time spent in questionnaire completion was 11.6±7.0 minutes.

### Follow-up outcomes

There was a slightly higher proportion of those who completed follow-up in the RA+ (90%, 155/173) compared with the RAO (82%, 125/152, OR 1.6, 95% CI 0.54–4.5, p = 0.39). Information about referral uptake and final diagnosis rates at 12 months are included in Table 2. Neither the referral recall nor uptake rates were significantly different between groups. Both the overall diagnoses and OSA diagnoses were significantly higher in the RA+ group. Approximately 16 patients would need to be screened (95% Confidence Interval (CI) for number needed to screen ranged from 9.3–67.0) with the flow monitor as part of the RA+ protocol to yield an additional confirmed diagnosis of OSA.

**Table 2:** Referral Results and Resulting Diagnoses in the two groups.

Outcome	RAO meeting criteria (Yes or +ve response)	RAO not meeting criteria (No or -ve response)	RA+ meeting criteria (Yes or +ve response)	RA+ not meeting criteria (No or -ve response)	OR (95% CI)	p-value,
Identified with any sleep disorder	82	70	136	37	3.22 (1.7–6.3)	<0.0001
Identified as at risk of OSA	54	98	114	5	3.40	0.001
Recall being referred at follow-up	41	83	58	97	1.30	0.40
Overall referral uptake	28	13	35	25	0.65	0.31
Referral recall in those at risk of OSA	20	24	49	55	1.10	0.81
Referral uptake in those at risk of OSA	12	42	28	86	1.01	0.97
Referral uptake in those who recall being referred	28	13	35	23	0.71	0.42
Referral uptake in those at risk of OSA and who recall being referred	12	8	28	21	0.87	0.81
Reported confirmed sleep disorder diagnosis at 12 months	6	146	17	156	2.65	0.03
Reported confirmed OSA diagnosis at 12 months	3	149	14	159	4.37	*0.01*

Note: OR and p values estimated using the Generalised Linear Mixed Models.

### Diagnostic characteristics of the screening tool: PSG sub-study

The PSG sub-study results for 20 randomly selected RA+ patients showed, with respect to a gold standard diagnosis of OSA defined as an apnea-hypopnea indeed of 5 or greater, that for the flow monitor (using the threshold of 18 events/hour), sensitivity was 0.78% (95% CI 0.40–0.96), specificity 0.67% (0.31–0.91), positive predictive value 0.70 (0.35–0.92), negative predictive value 0.75 (0.36–0.96) and the area under the receiver operating characteristic curve was 0.82. Thus two out of 8 participants who tested negative on the flow monitor were found to have OSA on their PSG. Using a threshold of 0.5 on the MAP index, we found a sensitivity of 0.22 (95% CI 0.04–0.60), specificity of 0.67 (0.31–0.91), positive predictive value 0.40 (0.07–0.83), negative predictive value 0.46 (0.20–0.74), and area under the ROC 0.37. Seven out of 13 patients (53%) who were classified as low risk of OSA on the MAPI were found to have OSA using their PSG. The study was not adequately powered to test for diagnostic accuracy differences between the two screening methods.

## Discussion

We compared the outcomes of two screening protocols for sleep disorders in the community pharmacy setting. As hypothesized, a protocol that includes an objective marker in the form of simple nasal flow monitoring, led to more patients being identified, referred and ultimately diagnosed with OSA, compared with one based on a questionnaire assessment only. A high proportion of pharmacy screened participants were at risk of a sleep disorder. Despite being given a written referral using a proforma referral letter, subject recall of being provided with referral was limited to a third of those referred irrespective of the method of screening. Actual recalled referral uptake was reported by a quarter of those who reported having been referred in either group. However of those participants who were referred, and who followed-up on the referral, a third were diagnosed with a sleep disorder. The results demonstrate the potential of community pharmacy as a novel primary healthcare venue where those ‘at risk of ’ and likely to have a common sleep disorder can be identified although methods of improvement in referral uptake need to be investigated.

Our hypothesis proposed a significant difference between the RAO group and the RA+ group, in terms of actual OSA diagnosis, as was the case. The rate at which participants identified as being at risk of OSA in the RA+ group took up their referral (by seeing their medical practitioner) was no higher than in the RAO group. Hence, the significantly higher rate of diagnosis in the RA+ group might be explained by a combination of a higher proportion being classified as at-risk and referred in the RA+ arm. An additional explanatory factor may be that the results from the objective sleep apnea test were more likely to influence the physician to initiate further investigations. Previous studies in other diseases have shown that the addition of a specific objective marker has enhanced the rate of diagnosis at the physician level [30]–[33].

Participant recall of referral was low, despite participants being provided computer generated referral letters which summarized the results to take to their primary care physician. Poor referral uptake has previously been shown in research conducted in the pharmacy setting [33] proving that our study is also reflective of ‘real-life’ clinical practice in which not all referred participants follow through on their referrals. Future allied health studies will need to be directed at improving referral uptake by participants, perhaps by strategies to trigger patient behaviour change, instituting pharmacist follow-up into the protocol, and enhancing direct collaboration and communication between the pharmacist and the primary care physician. In this study, while pharmacists informed physicians in their area about the research study and provided participants with referral letters, there was no other communication mandated by the protocol. Although the diagnostic yield from our study, in terms of the proportion of screened participants that were actually diagnosed, is low at 4% and 10% in the two groups, it is comparable to other pharmacy-based screening programs for conditions such as diabetes and cardiovascular disease that have a higher population prevalence than the targeted sleep disorders [1], [14], [31], [34]. This low diagnostic yield has occurred despite a targeted patient participant sampling approach that was based on symptoms, presence of comorbidities [35] or prescribed medications possibly associated with sleep disorders, that might potentially have recruited patients with a higher risk of sleep disorders than general pharmacy clients. Whether or not such a diagnostic yield leads to actual health and/or economic benefit is yet to be established in clinical studies. However, in the case of OSA, treatment has been shown to reduce health-related consequences caused by OSA [36]–[37]. While insomnia and RLS account for significant health and economic and economic costs [2], whether or not any identification of undetected cases of these conditions that results in successful treatment would lead to health and economic benefits would need to be tested.

Limitations in our study include the possibility of participation bias as participants self-selected for screening after seeing the recruitment materials. Furthermore, the randomisation at the pharmacy level may have led to selection bias in the RA+ group. In addition, the presence or absence of sleep disorders was only confirmed in those seeking further review (i.e. those referred by a pharmacist to their physician). Thus, we do not have robust estimates of the rate of false negatives, nor do we know the cost and morbidity borne by those who were false positive on screening. Furthermore, our results should be interpreted with caution as the lack of difference in rates of diagnoses for all conditions (besides OSA) between the two approaches may be the result of incomplete follow-up information. The proportion of participants identified as at risk of the sleep disorders considered in this study should not be interpreted as an estimate of the prevalence of these disorders in community pharmacy clients. Our choice to randomize the sample at the pharmacy level rather than the individual participant level may be critiqued as not a ‘true’ randomisation. However, we deemed cluster randomisation design as necessary in order to retain treatment fidelity and reduce treatment contamination within a pharmacy (where participants may have otherwise shared information).

In conclusion, pharmacy screening for sleep disorders is feasible and may lead to diagnosis of sleep disorders not previously diagnosed by medical practitioners. For obstructive sleep apnea specifically, our results suggest that a screening protocol which contains an objective marker improves the rate of receiving a confirmed sleep disorder diagnosis in a pharmacist-led screening and referral process. Future research needs to be conducted with protocols to establish cost effectiveness and evaluate treatment outcomes. The primary care screening model developed in this project has the capacity to improve the detection and management of people at risk of common sleep disorders, especially if referral uptake can be enhanced.

## Supporting Information

Checklist S1 **CONSORT Checklist.**
(DOC)

Protocol S1 **Trial Protocol.**
(PDF)

## References

[1] Committee on Sleep Medicine and Research (2006) Sleep Disorders and Sleep Deprivation: An Unmet Public Health Problem. Washington: Institute of Medicine.

[2] Deloitte Access Economics (2011) Re-awakening Australia. The economic cost of sleep disorders in Australia, 2010. Sleep Health Foundation

[3] Dikeos D, Georgantopoulos G (2011) Medical comorbidity of sleep disorders. Curr Opin Psych 24: 346–354. 10.1097/YCO.0b013e3283473375 21587079

[4] Skaer TL, Sclar DA (2010) Economic implications of sleep disorders. Pharmacoeconomics 28(11): 1015–1023. 20936885 10.2165/11537390-000000000-00000

[5] Gottlieb DJ, Yenokyan G, Newman AB, O'Connor GT, Punjabi NM, et al (2010) Prospective study of obstructive sleep apnea and incident coronary heart disease and heart failure: the sleep heart health study. Circulation 122: 352–360. 20625114 10.1161/CIRCULATIONAHA.109.901801 PMC3117288

[6] Young T, Finn L, Peppard PE, Szklo-Coxe M, Austin D, et al (2008) Sleep disordered breathing and mortality: eighteen-year follow-up of the Wisconsin sleep cohort. Sleep 31: 1071–1078. 18714778 PMC2542952

[7] Marshall NS, Wong KK, Liu PY, Cullen SRJ, Knuiman MW, et al (2008) Sleep apnea as an independent risk factor for all-cause mortality: the Busselton Health Study. Sleep 31: 1079–1085. 18714779 PMC2542953

[8] Laugsand LE, Vatten LJ, Platou C, Janszky I (2011) Insomnia and the Risk of Acute Myocardial Infarction: A Population Study. Circulation 124: 2073–2081. 22025601 10.1161/CIRCULATIONAHA.111.025858

[9] AlGhanim N, Comondore VR, Fleetham J, Marra CA, Ayas NT (2008) The Economic Impact of Obstructive Sleep Apnea. Lung 186: 7–12. 18066623 10.1007/s00408-007-9055-5

[10] Chai-Coetzer CL, Antic NA, Rowland LS, Catcheside PG, Esterman A, et al (2011) A simplified model of screening questionnaire and home monitoring for obshructive sleep apnoea in primary care. Thorax 66: 213–219. 21252389 10.1136/thx.2010.152801

[11] Bartlett DJ, Marshall NS, Williams A, Grunstein RR (2008) Predictors of primary medical care consultation for sleep disorders. Sleep Med 9: 857–864. 17980655 10.1016/j.sleep.2007.09.002

[12] Australian Government Department of Health and Ageing: Primary Health Care Reform in Australia - Report to support Australia's First National Primary Health Care Strategy. Element 3: More focussed on preventive care, including support of healthy lifestyles. Available: www.yourhealth.gov.au. Accessed 2011 December 15.

[13] Baraitser P, Pearce V, Holmes J, Horne N, Boynton PM (2007) Chlamydia testing in community pharmacies: evaluation of a feasibility pilot in south east London. Qual Saf Health Care 16: 303–307. 17693680 10.1136/qshc.2006.020883 PMC2464947

[14] Krass I, Mitchell B, Clarke P, Brillant M, Dienaar R, et al (2007) Pharmacy diabetes care program: Analysis of two screening methods for undiagnosed type 2 diabetes in Australian community pharmacy. Diabetes Res Clin Pract 75: 339–347. 16884811 10.1016/j.diabres.2006.06.022

[15] Mangum SA, Kraenow KR, Narducci WA (2003) Identifying at-risk patients through community pharmacy-based hypertension and stroke prevention screening projects. J Am Pharm 43: 50–55. 12585751

[16] Hersberger KE, Renggli VP, Nirkko AC, Mathis J, Schwegler K, et al (2006) Screening for sleep disorders in community pharmacies - evaluation of a c campaign in Switzerland. J Clin Pharm Ther 31: 35–41. 16476118 10.1111/j.1365-2710.2006.00698.x

[17] Tran A, Fuller JM, Wong KK, Krass I, Grunstein R, et al (2009) The devleopment of a sleep disorder screening program in Australian community pharmacies. Pharm World Sci 31: 473–480. 19462256 10.1007/s11096-009-9301-4

[18] Johns MW (1991) A new method for measuring daytime sleepiness: the Epworth Sleepiness Scale. Sleep 14: 540–545. 1798888 10.1093/sleep/14.6.540

[19] Bastein CH, Vallieres A, Morin CM (2001) Validation of the Insomnia Severity Index as an outcome measure for insomnia research. Sleep Med 2: 297–307. 11438246 10.1016/s1389-9457(00)00065-4

[20] Maislin G, Pack AI, Kribbs NB, Smith PL, Schwartz AR, et al (1995) A survey screen for prediction of sleep apnea. Sleep 18: 158–166. 7610311 10.1093/sleep/18.3.158

[21] Allen RP, Picchietti D, Hening WA, Trenkwalder C, Walters AS, et al (2003) Restless legs syndrome: diagnostic criteria, special considerations, and epidemiology. A report from the restless legs syndrome diagnosis and epidemiology workshop at the National Institutes of Health. Sleep Med 4: 101–119. 14592341 10.1016/s1389-9457(03)00010-8

[22] Sharwood LN, Elkington J, Stevenson M, Grunstein RR, Meuleners L, et al (2012) Assessing sleepiness and sleep disorders in Australian long-distance commercial vehicle drivers: self-report versus an “at home” monitoring device. Sleep 35: 469–475. 22467984 10.5665/sleep.1726 PMC3296788

[23] Rofail LM, Wong KKH, Unger G, Marks GB, Grunstein RR (2009) The utility of single nasal airflow pressure transducer in the diagnosis of OSA at home. Sleep 33: 1097–1105. 10.1093/sleep/33.8.1097 PMC2910540 20815193

[24] Crockett JA, Taylor SJ, McLeod LJ (2008) Patient responses to an integrated service, initiated by community pharmacists, for the prevention of osteoporosis. Int J Pharm Pract 16: 65–72.

[25] Effective Health Care Program, Comparative Effectiveness Review Number 32 (2011) Diagnosis and treatment of obstructive sleep apnea in adults, 2011. Agency for Healthcare Research and Quality, Dept of Health & Human Services, USA.

[26] Bland JM (2000) Sample size in guidelines trials. Fam Pract 17: S17–S20. 10735263 10.1093/fampra/17.suppl_1.s17

[27] R Development Core Team (2011) R: A language and environment for statitical computing. R Foundation for Statistical Computing, Vienna, Austria. ISBN 3-900051-07-0. Available: http://www.R-project.org/.

[28] The Pharmacy Guild of Australia (2009) Guild Digest.

[29] Hanes CA, Wong KK, Saini B (2014) Clinical services for obstructive sleep apnea patients in pharmacies: the Australian experience. Int J Clin Pharm. [Epub ahead of print] PubMed PMID: 24562977 10.1007/s11096-014-9926-9 24562977

[30] Maiman LA, Hildreth NG, Cox C, Greenland P (1992) Improving referral compliance after public cholesterol screening. Am J Public Health 82: 804–809. 1316721 10.2105/ajph.82.6.804 PMC1694199

[31] Snella KA, Canales AE, Irons BK, Sleeper-Irons RB, Villarreal MC, et al (2006) Pharmacy- and community-based screenings for diabetes and cardiovascular conditions in high-risk individuals. J Am Pharm Assoc 46: 370–377. 10.1331/154434506777069598 16739759

[32] Verne JE, Aubrey R, Love SB, Talbot IC, Northover JMA (1998) Population based randomized study of uptake and yield of screening by flexible sigmoidoscopy compared with screening by faecal occult blood testing. BMJ 317: 182–185. 9665902 10.1136/bmj.317.7152.182 PMC28612

[33] Allen H, Diamandis S, Saini B, Marshall D, Gavagna G, et al A Collaborative Screening, Referral and Management Process to Improve Health Outcomes in Chronic Obstructive Pulmonary Disease (COPD) report no. IIG052, The Pharmacy Guild of Australia, Canberra. Available: www.pharmacyguild.org.au. Accessed online 2012 August 23.

[34] Australian Institute of Health and Welfare, Australia's Health 2008. (2008) Canberra Australia.

[35] Alattar M, Harrington JJ, Mitchell MM, Sloane P (2007) Sleep Problems in Primary Care: A North Carolina Family Practice Research Network (NC-FP-RN) Study. J Am Board Fam Med 20: 365–374. 17615417 10.3122/jabfm.2007.04.060153

[36] Marin JM, Carrizo SJ, Vincente E, Agusti AGN (2005) Long-term cardiovascular outcomes in men with obstructive sleep apnoea-hypopnea with or without treatment with continuous positive airway pressure: an observational study. Lancet 365: 1046–1053. 15781100 10.1016/S0140-6736(05)71141-7

[37] George CF (2001) Reduction in motor vehicle collisions following treatment of sleep apnoea with a nasal CPAP. Thorax 56: 508–512. 11413347 10.1136/thorax.56.7.508 PMC1746094

