# Supplementary material for: A Comparison of Screening Methods for Sleep Disorders in Australian Community Pharmacies: A Randomized Controlled Trial
Source: PLoS One. 2014 Jun 30;9(6):e101003. doi: 10.1371/journal.pone.0101003 (PMC4076224; doi:10.1371/journal.pone.0101003)
Supplement: Protocol S1 — Trial Protocol. (PDF) [file pone.0101003.s002.pdf]

ETHICS AND PRIVACY APPLICATION FORM FOR RESEARCH INVOLVING HUMANS

Please Note: Each question on this form has instructions and links to relevant documents and guidelines on how to answer that particular question as hidden text. To show the text with the hidden text effect, click symbol "¶" (**Show/Hide)** (situated next to the **"Zoom"** button) on the "**Standard"** toolbar. When hidden text is shown it is marked with a dotted underline. This text will not be seen on the printed version.

Please note the following:

1. This application must be completed electronically or typewritten
2. Complete all sections except those specifically not applicable
3. Use lay terms wherever possible
4. Do not alter the order of questions or layout of the application form
5. "Y" signifies Yes, "N" signifies No, and "N/A" signifies Not applicable
6. Some "Y"/"N" boxes have been reversed so take care in answering the questions
7. HREC refers to Human Research Ethics Committee

SECTIONS:

		Page
Section 1:	Administration	2
Section 2:	Nature of Research	6
Section 3:	Participants and Recruitment	7
Section 4:	Privacy	10
Section 5:	Collection and Dissemination of Results	13
Section 6:	Risks and Benefits	15
Section 7:	Participant Information and Consent	16
Section 8:	Conflict of Interest and Other Ethical Issues	17
Section 9:	Description of Project	18
Section 10:	Field Based Research or Research Conducted Outside Australia	20
Section 11:	Research Involving Blood, Tissue, etc.	22
Section 12:	Clinical Trials of Drugs or Devices	24
Section 13:	Declaration of Researchers	25
	Checklist	26

This form has been prepared in collaboration between Ms G Briody, Associate Professor M Grimm, Professor A Lloyd, Associate Professor J Watson and Ms M Wright of the Human Research Ethics Committees (HRECs) of the Universities of New South Wales and Sydney.

SECTION 1: ADMINISTRATION

This section is obligatory

1.1 (a) Full project title

Role of pharmacists in sleep health –a screening, awareness and monitoring program

(b) Short name by which the project will be known

Pharmacists and Sleep Health

(c) Name of Chief Investigator

Dr Bandana Saini

(d) Provide a brief summary of the project in lay language (approximately 100 words)

Sleep disorders are a significant public health issue in Australia. Pharmacists are health professionals who have frequent contact with patients who may be at risk of sleep disorders or already have an existing sleep problem. This project aims to develop an online **basic** screening program that specially trained pharmacists can use on their dispensing computers to screen willing clients/patients about their risk of having a sleep disorder. Pharmacists will be randomised into two groups. In the control group, pharmacists will run the **basic** screening program, identify and refer patients at risk and follow up after 3 months to document patient's actions and referral consequences. In the intervention arm, patients who are identified through the online screening tool as being at higher risk will be invited to participate in a **comprehensive** screening program involving the use of valid sleep health questionnaires, sleep diaries and a nasal device that can provide information about nasal airflow during sleep and can be used by patients themselves at home. 'At risk' patients will be referred to physicians and appropriate follow up conducted after 3 months to test the comparative effectiveness of the basic and comprehensive screening tools developed through this project.

(e) Outline the scientific merits of this study (including potential contributions to the body of knowledge and methodological rigor) (approximately 100 words)

This will be the first project to test the effectiveness of community pharmacies as an alternative health care site for the improvement of sleep health outcomes in Australia, and one of the first globally. Through the participation of pharmacists, patients with sleep disorders will have improved access to information about the diagnosis, treatment and management of their conditions, especially in rural settings. The increased awareness and education about sleep health amongst pharmacists and the public will allow for improved identification of sleep disorders and disease management, and lead to cost savings for patients, providers and the health care system. Data collected from the study will help shape future models for sleep disorders screening in primary health care sites.

1.2 Indicate the institutional ethics committee that you consider to be the primary one for this project. (In general, if the Chief Investigator is a University employee, then the University should be considered to be the primary site. If the Chief Investigator or participants are from a health care service, then the Area Health Service ethics committee should be considered as the primary site.)

University of Sydney

1.3 (a) Has this project already been submitted to any other HREC(s)? ☒ **N** ☐ **Y**

(b) Will this project be submitted to any other HREC(s)? ☒ **N** ☐ **Y**

If you answered YES to (a) or (b), give the name of the HREC(s), and indicate the status of the application at each (i.e., submitted, approved, deferred or rejected). Attach copies of the correspondence with each of the other HREC(s).
Please do not submit to more than one HREC <u>concurrently</u>.

1.4 List the following details of the Chief Investigator/Supervisor, any Co-Researcher(s), Associate Researcher(s) and Student(s).

Chief Investigator/Supervisor

Name	**Bandana Saini**
Title	Dr
Qualifications	BPharm., MPharm., MBA, PhD, Grad Cert Higher Edu
Positions held: employed, conjoint/adjunct/visiting	Lecturer, Pharmacy Practice, Faculty of Pharmacy, University of Sydney
Full mailing address (including building number)	Building A15, Science Rd, Faculty of Pharmacy, University of Sydney, Camperdown, NSW 2006
Telephone	02 93516789
Fax	02 93514391
E-mail	bandana@pharm.usyd.edu.au

Co-Researcher(s), Associate Researcher(s), Student(s) or other Personnel involved in the study (If appropriate indicate for each named person whether they are University staff, student or neither). If the named person is a student, nominate (in the Qualifications section) the degree for which he/she is enrolled.

Name	**Keith Wong**
Title	Dr
Qualifications	MBBS, FRACP
Positions held: employed, conjoint/adjunct/visiting	Research Fellow, Woolcock Institute of Medical Research
Full mailing address (including building number)	Woolcock Institute of Medical Research, PO Box M77, Camperdown NSW 2050
Telephone	Tel: 02 9515 6691
Fax	Fax: 02 9515 7070
E-mail	keithw@med.usyd.edu.au

Name	**Ines Krass**
Title	A/Prof
Qualifications	
Positions held: employed, conjoint/adjunct/visiting	Head, Pharmacy Practice Group, Faculty of Pharmacy, University of Sydney
Full mailing address (including building number)	Building A15, Science Rd, Faculty of Pharmacy, University of Sydney, Camperdown, NSW 2006
Telephone	02 93513507
Fax	02 93514391
E-mail	inesk@pharm.usyd.edu.au

Name	**Ron Grunstein**
Title	Professor
Qualifications	MBBS, MD, PhD, FRACP
Positions held: employed, conjoint/adjunct/visiting	*Clinical Professor*<br>Medicine, Central Clinical School, University of Sydney
Full mailing address (including building number)	C39 - Royal Prince Alfred Hospital, The University of Sydney, NSW 2006
Telephone	+61 2 9515 8630
Fax	+61 2 9515 7070
E-mail	rrg@med.usyd.edu.au

Name	***A project officer will be hired to work on the project. There is also a possibility that a B.Pharmacy final year (Advanced stream) student may work on elements of the project. The names of these research investigators will be submitted as soon as they become available for addition on to the project team.***
Title	
Qualifications	
Positions held: employed, conjoint/adjunct/visiting	
Full mailing address (including building number)	
Telephone	
Fax	
E-mail	

Insert additional boxes if necessary.

1.5 Who is the nominated Contact Person (from those listed in 1.4 above) for this protocol?

Name	Telephone Number	Email
Bandana Saini	02 93516789	bandana@pharm.usyd.edu.au

1.6 Who is the person preparing this document?

Name	Telephone Number	Email
Bandana Saini	02 93516789	bandana@pharm.usyd.edu.au

1.7 In addition to the researchers named in 1.4 are there students involved as researchers in this project? ☐ N ☑ Y

Not sure- elements of this project will be offered to B.Pharm final year students (Advanced Stream) and depends on them opting for the project as their choice for working on in their advanced year.
Only 1 honours student will be allocated to work on the project.

If you answered YES, indicate the number of students covered by this study and the degrees which this study will contribute towards (i.e., Honours, Masters, PhD, etc.) **If the names are already known please include them.**

One student – B.Pharm, Year 4th Advanced Stream (equivalent to honours)

1.8 (a) Indicate the proposed date of commencement of the project.
Projects may not commence without the prior written approval of the HREC.

Date April 2008

(b) Indicate the proposed completion date of the project.

Date December 2009

1.9 Indicate all location(s) at which the research will be undertaken.

The primary location will be University of Sydney- where all developmental work will be undertaken. Once a screening tool is developed, pharmacists in NSW/ACT will be recruited to participate. This pharmacist will then conduct research (screening of patients, documenting data, referring patients) in their pharmacies.

1.10 (a) Has this protocol received research funding/contracting or is this submission being made as part of an application for research funding/contracting? ☐ N ☑ Y

If you answered YES, list the funding/contracting bodies to which you have submitted, or intend to submit, this project. Attach a copy of the grant application(s), contract(s) or similar agreement(s).

Funding/Contracting body 1: The Pharmacy Guild of Australia, Investigator Initiated Grants Scheme 2007
Funding/Contracting body 2:
Funding/Contracting body 3:

(b) What is the outcome of these funding/contracting application(s) (please tick the appropriate box)

	Approved	Pending	Refused
**Funding/Contracting body 1:**	☑	☐	☐
**Funding/Contracting body 2:**	☐	☐	☐
**Funding/Contracting body 3:**	☐	☐	☐

(c) Will this study still be undertaken if funding is not successful?

NA: pending contract confirmation, the funding has been approved

☐	☐
**N**	**Y**

(d) If the title of the project submitted for funding is different from that listed under Q1.1(a), state it below.

**NA**

Proceed to Section 2.

SECTION 2: NATURE OF RESEARCH

(refer to the National Statement on Ethical Conduct in Research Involving Humans, p. 23-45)

This section is obligatory

2.1 The nature of this project is most appropriately described as research involving:- (more than one may apply):

	N	Y
– behavioural observation	☒	☐
– self-report questionnaire(s)	☐	☑
– interview(s)	☒	☐
– qualitative methodologies (e.g. focus groups)	☒	☐
– psychological experiments	☒	☐
– epidemiological studies	☒	☐
– data linkage studies	☒	☐
– psychiatric or clinical psychology studies	☒	☐
– human physiological investigation(s)	☒	☐
– biomechanical device(s)*	☐	☑
– human tissue (see Section 11)	☒	☐
– human genetic analysis (see Section 11)	☒	☐
– a clinical trial of drug(s) or device(s) (see Section 12)	☒	☐
– Other (please specify in the box below)	☐	☐

*The basic screening will consist of asking patients to answer questions based on validated sleep questionnaires. Patients who are identified as being at risk of a sleep disorder in the intervention arm of the study will undergo a comprehensive screening beyond the basic screening. This comprehensive screening will consist of patients recording a sleep diary and using a mechanical device that monitors nasal airflow whilst they sleep. The nasal airflow monitor selected for the purposes of this study (Flow Wizard, DiagnoseIT, Sydney, New South Wales, Australia) consists of both a recording device, and analysis software. The recorder (dimensions 11.5 cm by 7 cm by 4.2 cm, 148 grams including batteries) employs a pressure transducer, analogue to digital conversion and storage hardware to record the nasal airflow collected by means of standard nasal oxygen cannulae. The software provided with the device employs an algorithm to analyse characteristics including the amplitude and stability of the flow/pressure curve to distinguish snoring and respiratory events from normal breathing. The results from this software will be available to the pharmacist, who will interpret the readings and prepare a brief report for the physician. This device was developed and is patented through research at the Woolcock Institute of Medical Research (*Unger, G., Hedner, J. A., Grunstein, R. R., & Williams, A. (2003). Diagnostic accuracy of a single channel nasal pressure recording in patients referred for investigation of sleep apnea. Sleep, 26, A401.*) .
Whilst Polysomnography is the gold standard test for the diagnosis of sleep disorders such as sleep apnea, the FlowWizard is much less expensive and less complicated device that can be used to detect disordered breathing, and data used to exclude/suspect sleep complications.
Similar devices are also available from different companies and widely advertised on the internet for home use by people who believe they may be at risk of having a sleep apnea

Proceed to Section 3.

SECTION 3: PARTICIPANTS AND RECRUITMENT
(refer to the National Statement on Ethical Conduct in Research Involving Humans, p. 25-34)

This section is obligatory

3.1 (a) What is the age range of all participants involved in this study?

18 and above

(b) If the participants include children (defined by statute for this purpose as anyone under 18) has a Prohibited Employment Declaration Form for the researchers ("criminal record check") been lodged with the University or hospital? (see http://www.kids.nsw.gov.au/check/) ☐ Y ☐ N

If you answered NO, give reasons why not.

NA

3.2 Are the participants:-
(more than one may apply)

	N	Y
– in a teacher–student relationship with the researchers or their associates?	☒	☐
– in an employer–employee relationship with the researchers or their associates?	☒	☐
– in any other dependent relationship with the researchers or their associates?	☒	☐
– wards of the state?	☒	☐
– prisoners?	☒	☐
– refugees?	☒	☐
– members of the armed services?	☒	☐
– mentally ill?	☒	☐
– intellectually impaired?	☒	☐
– unconscious or critically ill patients?	☒	☐
– under the Guardianship Act 1987 (as amended)?	☒	☐
– in a doctor–patient relationship or a health giver–receiver relationship with the researchers or their associates?	☒	☐
– Aboriginal or Torres Strait Islanders?	☒	☐

If you answered YES to any of the above, provide details.

3.3 (a) What is the sample size for the study? Comment on how this sample size will allow the aims of the study to be achieved.

Basic screening arm
In a Swiss study*, an online instrument based screening program identified about 26% of screened patients who were 'at risk' for sleep disorders and were referred to the physician. We will use a conservative estimate in our study, i.e. 10% of screened patients will be identifiable as being 'at risk' using an online screening instrument (**i.e. the basic screening**). Using a power of 90% and two sided confidence intervals of 5%, the sample size required to be screened to detect 'at risk' patients, is 48.

Comprehensive screening arm
It is assumed that within the comprehensive screening program, the proportion of patients identified and referred will be equivalent to the Swiss study, i.e. 26%.21 Using a 90% power and a 5% confidence interval, to detect a 16%(26%-10%) difference of referral rate between the basic and comprehensive screening group, a minimum 114 patients in each arm will be required.

Total required- Combining the sample size requirements from above, and allowing for a cluster effect and loss to follow up phenomena, it can be calculated that a minimum of 200 patients are required to be screened.

*****_Hersberger KE. Renggli VP. Nirkko AC. Mathis J. Schwegler K. Bloch KE. Screening for sleep disorders in community pharmacies--evaluation of a campaign in Switzerland. Journal of Clinical Pharmacy & Therapeutics. 31(1):35-41, 2006 Feb._

(b) How will the participants be recruited?

PARTICIPATING PHARMACISTS
Expressions of interest will be sought from pharmacists who specialise in sleep health and related products. These pharmacies/pharmacists will be identified based on lists obtained from manufacturers/suppliers for pharmacies that serve as outlets for sleep related devices, and 20 pharmacists recruited across NSW and ACT(information on pharmacies that supply sleep health product is readily available from sleep clinics and the internet, and is therefore a public domain). If this number is not achieved from this group, recruitment will be opened to general pharmacists, selected from a list of pharmacies accredited through the Pharmacy Guild of Australia's Quality Care Pharmacy Program (QCPP) in NSW/ACT. These selected QCPP pharmacies will be approached until the target number is achieved. Within the sample, pharmacies will be matched and paired, and in each matched pair pharmacies will be randomly assigned to either the:
1) **Intervention** (comprehensive sleep screening for high risk patients) or
2) **Control** (basic sleep screening only) group.

Following training, all pharmacists will be provided with promotional materials. Posters will target people with symptoms and risk factors for sleep disorders, and invite them to talk to their pharmacist. Collaborative initiatives between pharmacy and other health care professionals will be set up at this stage. The Sleep Awareness Campaign will be run for three months Pharmacists will recruit patients who make enquiries whilst the Sleep Awareness Campaign is running. ***To reach the target sample size, each pharmacist will be requested to screen and complete documentation for at least 20 patients, (20 pharmacies x 20 patients = total 400 patients screened, 200 in each group).***

PARTICIPATING PATIENTS
If recruitment targets have not been met through patient's responding to the promotional phase, pharmacists will also be asked to approach their adult patients who have a dispensed medication history for any medications related to cardiovascular symptoms, diabetes, asthma, rhinitis or COPD (a checklist of medications will be provided to pharmacists). Patients inquiring about products to help fatigue and energy levels or weight reduction will also be invited to participate in the study. Patients already with a diagnosis of a sleep problem, or those < 18 years of age, or those not fluent enough to understand and answer the screening questions without the aid of a translator, or those with terminal/debilitating illness will not be included in the study.

3.4 (a) Does recruitment involve a direct personal approach from the researchers to the potential participants? [x] **N** [ ] **Y**

If you answered YES, explain how the real, or perceived, coercion from researchers for potential participants to enrol has been addressed.

There will be no direct contact between the researchers and patients.
Researchers will approach pharmacists – who in turn will recruit their patients for participation in the study.

Pharmacists -approach for participation process
As mentioned above, lists of pharmacies that supply medical equipment related to sleep health (e.g. Continuous Positive Airways Pressure machines); will be obtained from major manufacturers/suppliers of such equipment. Pharmacies will be sent an expression of interest about the project (fax or mail). If there is a response, the project officer will ring the pharmacies to expand on the aims of the project and what participation entails. Pharmacies that do not respond will be rung after a few weeks to see if they have had a chance to read the flyer and the possibility of participation. If sufficient pharmacies are not obtained in this first round of recruitment, then a list of all pharmacies that have been accredited within the Quality Care Pharmacy Program in NSW will be sought from the Pharmacy Board of NSW. Pharmacies in areas that have had none of the sleep specialist pharmacists respond, or where there are no specialist pharmacies will be targeted first from this list. The same process for recruitment will be followed –e.g. provision of information about the project and invitation of those pharmacists interested in participating and one telephone follow up in case of non-response. This process does not involve face-to-face meeting of the researchers with the pharmacists. **The expression of interest will clearly state that participation in the project is voluntary and can be withdrawn at any stage.** Any project officers conducting the recruitment follow up calls will be trained to re-inforce this message.

(b) Does recruitment involve the circulation/publication of an advertisement, circular, letter, etc? ☐ N ☑ Y

If you answered YES, provide a copy and indicate where and how often it will be published.

The expression of interest to participate in the project will be sent to pharmacies identified as those specialising in sleep health. If an insufficient number of pharmacists are recruited into the study, other recruitment strategies will include- distributing the letter of invitation to participate in the project the Pharmaceutical Society of Australia(PSA), NSW branch will be requested to display a copy of the letter at Professional Development Days for pharmacists that are conducted through the society(this will be done by PSA personnel, if the PSA agrees, and so there is no direct face to face approach from the researchers).

3.5 Will participants receive any reimbursement of out-of-pocket expenses, or financial or other "rewards" as a result of participation? ☐ N ☐ Y

If you answered YES, what is the amount or nature of the reward and the justification for this?

Patients in the control arm will receive a $10 ***voucher*** and those in the intervention arm will receive a $25 ***voucher*** to spend on health related products in the pharmacy, as a small token of appreciation of their time. Pharmacists who participate in the project will expend a considerable amount of time training in sleep health, and prioritizing project activities such as handling enquiries about sleep health ensuing because of the Sleep Health Awareness. These pharmacists will be paid for 8 hours of training (for which they may need to hire a locum pharmacist whilst they attend training), and either $15 per patient (basic screening) or $60(comprehensive screening). This payment is not likely to be commensurate with the amount of time spent by pharmacists in recruiting patients, setting up appointments, and then actually screening patients, however is set up in the project, so that pharmacist participants have some recompense for their time and effort, and also to bring a touch of 'realism', where in the future such screening may be a marketable service provided by pharmacies (and funded by the Commonwealth or private health insurance or patient payment etc).

3.6 Is the research targeting any particular ethnic or community group? ☒ N ☐ Y

If you answered YES, which group is being targeted?

NA

If you answered YES, is there an investigator who is a member of the Particular ethnic or community group? ☐ Y ☐ N

If you answered YES to 3.6, has this project been planned in consultation with a representative of this group? ☐ Y ☐ N

If you answered YES, who have you consulted and how do they represent this group?

NA

If you answered NO, give reasons why you have not consulted.

NA

Proceed to Section 4.

SECTION 4: PRIVACY
Refer to the National Statement on Ethical Conduct in Research Involving Humans, p. 52-53. For health related information refer to the Statutory Guidelines made under the *Health Records and Information Privacy (HRIP) Act 2002 (NSW) Statutory Guidelines on Research* via Privacy NSW HRIP Act and also the NHMRC overview document *The Regulation of Health Information Privacy in Australia* http://www.nhmrc.gov.au/publications/synopses/nh53syn.htm

This section is obligatory

4.1 Is there a requirement for the researchers to identify, collect, use, or disclose information of a personal nature *(either identifiable or potentially identifiable)* about individuals without their consent?

		N	Y
**(a)**	**from Commonwealth departments or agencies?**	[x]	[ ]
**(b)**	**from State departments or agencies?**	[x]	[ ]
**(c)**	**from other third parties, such as non-government organisations?**	[x]	[ ]

If you answered YES to (a), (b) or (c), state what information will be sought and how many records will be accessed.

NA

			N	Y
**4.2**	**(a)**	**Is there a requirement for the researchers to identify, collect, use, or disclose personal health information about individuals without their consent, which is identifiable or potentially identifiable?**	[x]	[ ]

IF YOU ANSWERED NO, YOU DO NOT NEED TO COMPLETE ANY MORE OF SECTION 4. GO TO SECTION 5

If you answered YES, indicate the reason(s)

- The project involves linkage of data [ ] Y
- Scientific deficiencies would result if de-identified information was used [ ] Y
- Other [ ] Y

Please provide details

4.3 Will the health information that is identifiable or potentially identifiable with respect to individuals be collected, used or disclosed without the consent of the individual(s) concerned? [ ] N [ ] Y

If you answered YES, indicate the reason(s)

- The size of the population involved in the research. [ ] Y
- The proportion of subjects who are likely to have moved or died since the health Information was originally collected. [ ] Y

– The risk of introducing bias into the research, affecting the generalisability and validity of the results. ☐ Y

– The risk of creating additional threats to privacy by having to link information in order to locate and contact subjects to seek their consent of the results. ☐ Y

– The risk of inflicting psychological, social or other harm by contacting subjects with particular conditions in certain circumstances. ☐ Y

– The difficulty of contacting individuals directly when there is no existing or continuing relationship between the organisation and the individuals. ☐ Y

– The difficulty of contacting individuals indirectly through public means, such as advertisement and notices. ☐ Y

– Other ☐ Y

Please provide details

4.4 Was this research the primary purpose of collecting the health information? NA ☐ **Y** ☐ **N**

If you answered YES, you do not need to complete any further questions in Section 4. Go to Section 5

If you answered NO, please provide details

4.5 Would the subjects have expected the researchers to use or disclose their health information for the purposes of this project? ☐ **Y** ☐ **N**

Please provide details

4.6 Explain why the collection, use or disclosure of this information is in the public interest, and why the public interest in the project substantially outweighs the public interest in the protection of privacy.

Proceed to Section 5.

SECTION 5: COLLECTION OF DATA AND DISSEMINATION OF RESULTS
(refer to the National Statement on Ethical Conduct in Research Involving Humans, p. 52-53)

This section is obligatory

5.1 Will any part of the study involve recordings using audio tape, film/video, or other electronic medium ? ☐ N ☑ Y

If you answered YES, what is the medium and how it will be used?

The screening tool developed to identify patients at risk of developing screen disorders will be converted to an online version-POTASH(Pharmacy Online Tool for the Assessment of Sleep Health). This tool will enable pharmacists to enter patient variables and have a 'risk' of sleep disorder score automatically calculated.

5.2 Does your research involve the secretive use of photographs, tape-recordings, or any other form of record-taking? ☒ N ☐ Y

If you answered YES, provide details and a justification for the secrecy.

NA

5.3 (a) How will the results of the study be disseminated (e.g. via publication in journals and presentations in scientific meetings)?

The project's setup, methods, process and outcome results will be collated in the form of a report at the end of the project. The report will be sent to the Pharmacy Guild of Australia. The results of the study may also be written as a manuscript for publication in a peer-reviewed journal. The key results will also be disseminated at professional conferences and scientific meetings.

(b) How will feedback be made available to participants (e.g. via a lay summary or newsletter)?

The project results will be written in the form of a report that will be submitted to the Pharmacy Guild of Australia (Funding Body). An executive summary of the report will be made available to all the participants. A debrief meeting will be held at the end of the project to provide information on project processes and outcomes to the pharmacists. At this meeting, pharmacist feedback about the utility and feasibility of the screening service will be invited and responses (de-identified) collated in the final report.

5.4 How will the confidentiality of the data, including the identity of participants, be ensured during collection and dissemination?

All participating pharmacists will be requested to sign a consent form before commencing participation. All consent forms will be coded, and once signed, any data entry involving a particular participating pharmacist will utilise the code, so no names or initials are entered into the databases.

All pharmacies and participating pharmacists will be issued patient consent forms and information sheets. These sheets will also have a code at the bottom. Once a patient consents, this code will be used to identify the patients in the database- so that no names or initials are entered into the online database. The online screening program itself will be password protected, and will be placed on pharmacy dispensing computers to which only pharmacists usually have access. Pharmacists will be provided with a brief training on confidentiality issues and managing confidentiality of patient data at their workplaces. Once the screening phase is over, the data will be electronically transferred by a project officer into a statistical package (SPSS) maintained at the University. Patient data will be deleted from pharmacy computers, once it has been successfully transferred to the SPSS database. Only the project officer and research team members will have access to the database.

5.5 Is there any possibility that information of a personal nature could be revealed to persons not directly connected with this research? ☒ N ☐ Y

If you answered YES, provide details.

5.6 (a) What is the proposed storage location of, and access to, materials collected during the study (including files, audiotapes, questionnaires, videotapes, photographs)?

Data collected during the project will consist of questionnaires, electronic databases, and audiotapes.
Pharmacists will enter de-identified (as explained in 5.4 above) patient data onto a password-protected program on their dispensary computers. This data will be transferred electronically to a database maintained at a location within the Faculty of Pharmacy, University of Sydney. Any 'hard copy' data collected by and from pharmacists will be collected and filed by the project officer. This data will be placed in a filing cabinet that is locked with access only to the researchers for the duration of the project. Electronic databases will be maintained on a single computer that will be used by a project officer- it will be ensured this computer is also password protected.
At the completion of the project, all electronic items will be deleted from the hard drive of the computer terminals being used and transferred to USB sticks, which will be maintained by the Chief Investigator in a secure cabinet to which only they will have access. All hard copy materials and audiotapes will be stored in sealed archive boxes with the project label and placed in the limited access 'Archive' area within the Faculty of Pharmacy. Both the USB Sticks and the archive boxes will be kept for the stipulated period of seven years.

(b) Specify how long materials collected during the study (including files, audiotapes, questionnaires, videotapes, photographs) will be retained after the study, and how they will ultimately be disposed of.

Please ensure that the period of data retention stated here is appropriate to the nature of the proposed study. If the project involves clinical trial(s), the data should be kept for a minimum of 15 years (please refer to http://www.fda.gov/oc/ohrt/irbs/websites.html). If the projects do not involve clinical trial(s), the data should be kept for a minimum of 7 years after which time the data may be disposed of. *(Please also refer to National Statement on Ethical Conduct in Research Involving Humans, 12.11 for further requirements*).

As mentioned above, both the USB sticks (with soft copy data) and the archive boxes (hard copy date) will be kept for the stipulated period of seven years. After a period of seven years, all hard copy materials will be incinerated. De-identified data on USB devices will be maintained if further research on sleep health and the role of pharmacists and other primary care practitioners is still being conducted.

Proceed to Section 6.

SECTION 6: RISKS AND BENEFITS
(refer to the National Statement on Ethical Conduct in Research Involving Humans, p. 51)

This section is obligatory

6.1 (a) Could participation in the research adversely affect the participants? [x] N [ ] Y

If you answered YES, complete 6.1 (b) and 6.1 (c). If you answered NO go to 6.2

(b) Could the research induce any psychological distress in the participants? [ ] N [✓] Y

(c) Could the research cause any physical harm to the participants?
(e.g. from physically invasive procedures or from drug administration, etc) [x] N [ ] Y

If you answered YES to (b) or (c) describe the aspect(s) of the research and all the risks involved. Indicate the rate at which these risks are expected to occur. Indicate what facilities and trained personnel are available to deal with such psychological or physical problems.

Patients who consent to be screened may experience apprehension about their chances of having or developing a sleep disorder. The level of this apprehension may be the same as when any person visits a doctor's surgery for an annual check up. It is expected that in three fourths of the cases, this anxiety can be dispelled within a few minutes as the pharmacists translate the results of the screening into a lay language for those not identified as having or developing a sleep disorder. For those at risk, pharmacists will be trained to use an approach that will help dispel anxiety and encourage patients to seek further assistance from physicians.

6.2 Will the true purpose of the research be concealed from the participants? [x] N [ ] Y

If you answered YES, outline the rationale and provide details for the concealment.
Provide details of the debriefing. (If you do not intend to debrief, give reasons why not).

NA

6.3 Are you doing research on patients (i.e. subjects receiving health care)? [ ] N [✓] Y

If you answered YES, list the procedures/techniques which would <u>not form part of routine</u> clinical management.

Basic screening- the control arm
The screening questionnaire that will be developed in the project will mainly consist of health related questions such as age, weight, smoking history, sleeping patterns, medical history etc. Whilst **all** these questions may not be asked by pharmacists of their patients at a single encounter normally, they often do form part of normal pharmacovigilance and pharmaceutical care activities, which are the professional duty of the pharmacist. Pharmacists often ask such questions when patients are on certain medications that affect sleep or when patients query about over the counter products for aiding sleep. Often pharmacists record pertinent notes about a patient's health or over the counter medicine use in their dispensing database as part of good dispensing practice. Therefore, in the basic screening arm, the procedures that will form part of the project protocol are not vastly different from normal practice.

Comprehensive screening- the intervention arm
For patients identified by pharmacists in the intervention group pharmacists as being at risk, a more comprehensive screening is envisaged. This comprehensive screening will consist of patients keeping a sleep log, and using a nasal airflow monitoring device that measures nasal airflow during sleep. Whilst the gold standard of diagnosing sleep disorders is usually a full scale polysomnography(PSG) test conducted in specialised sleep laboratories, the PSG is time consuming, labour intensive and disruptive to normal routine in patients. An alternative method to check for the possibility of obstructive sleep apnoea or sleep disordered breathing patterns is to use a device that measures the airflow through the nose while a person sleeps. Such devices are simple, and can be used by patients at home.
Study protocol- Within this study, consenting patients at risk of sleep disorders in the intervention arm, will be asked to wear the nasal cannulae of the flow monitor upon retiring to bed, and to press a button on the device that triggers the recording of the signal for nine hours(or for the duration of their sleep). The recording will be performed for three consecutive nights at home, before returning the device for downloading to the pharmacy. The software provided with such devices employs an algorithm to analyse characteristics including the amplitude and stability of the flow/pressure curve to distinguish snoring and respiratory events from normal breathing. Pharmacists in the intervention arm will supplement data from patient's sleep logs and the nasal airflow devices and refer patients to their GPs with the appropriate notes and summary of results for further testing and diagnosis.

6.4 Is this research expected to benefit the participants directly or indirectly?

☐ N ☑ Y

If you answered YES, provide details.

Participating patients who may have a risk of having or being affected by a sleep disorder will obtain guidance and counseling about further action from the trained participating pharmacist. Those not at risk will obtain greater awareness about sleep health, so that they can minimise their risks in the future.

Participating pharmacists will gain a sense of professional fulfillment. Performing a screening service may enhance their professional image in the eyes of their clientele and lead to patient loyalty and trust.

Proceed to Section 7.

SECTION 7: PARTICIPANT INFORMATION AND CONSENT
(refer to the National Statement on Ethical Conduct in Research Involving Humans, p.12-13, p.28-29, p. 40-42, p.44-45, p.47-50, p.54)

This section is obligatory

7.1 Will a Participant Information Statement be provided? ☑ Y ☐ N

7.2 Will written consent be obtained? ☑ Y ☐ N

If you answered NO to either 7.1 or 7.2, give reasons why not.

7.3 In the case of participants who may not be fluent in English or who have difficulty understanding English, will arrangements be made to ensure comprehension of the Participant Information Statement and Consent Form? ☐ Y ☒ N

If you answered NO, give reasons. If you answered YES, what arrangements have been made?

Pharmacists are practicing health professionals expected to be fluent in the English Language. Patients who are unable to comprehend the language sufficiently to answer the screening questions will not be recruited in the study.

7.4 (a) Do the Participant Information Statement and Consent Form have:-

- **the first page of the Participant Information Statement and Consent Form printed on appropriate institutional letterhead?** ☑ Y ☐ N
- **the title of the project on every page, including the Revocation of Consent? (if one is required) (Use a short title as appropriate)** ☑ Y ☐ N
- **the page numbers expressed as page 1 of .., 2 of .., 3 of .. etc?** ☑ Y ☐ N
- **an assurance that participation is voluntary and participants are permitted to withdraw from the project at any time without penalty?** ☑ Y ☐ N
- **the name and telephone number of an appropriate researcher?** ☑ Y ☐ N
- **a telephone number, fax number and E-mail address for the HREC, should a participant wish to make a complaint about the conduct of the research project?** ☑ Y ☐ N

(b) How has the possibility of withdrawal from the study been addressed in the Participant Information Statement and Consent Form?

Proceed to Section 8.

SECTION 8: CONFLICT OF INTEREST AND OTHER ETHICAL ISSUES
(refer to the National Statement on Ethical Conduct in Research Involving Humans, p. 51–54, Appendix 2)

This section is obligatory

8.1 Are any "conflict of interest" issues likely to arise in relation to this research? N [×] Y [ ]

If you answered YES, provide details.

8.2 Do the researchers have any affiliation with, or financial involvement in, any organisation or entity with direct or indirect interests in the subject matter or materials of this research? N [×] Y [ ]
(Note that such benefits must be declared in the Participant Information Statement.)
If you answered YES, provide details.

8.3 Do the researchers expect to obtain any direct or indirect financial or other benefits from conducting this research? N [×] Y [ ]
(Note that such benefits must be declared in the Participant Information Statement.)
If you answered YES, provide details.

8.4 (a) Have conditions already been imposed upon the use (eg. publication), or ownership of the results (eg. scientific presentations) or materials (eg. audio-recordings), by any party other than the listed researchers? N [ ] Y [✓]

(b) Are such conditions likely to be imposed in the future? N [ ] Y [✓]

If you answered YES to (a) or (b), provide details.

At this stage, the funding body imposes no limitations on the researchers towards publication of the research and for use of materials developed through the research for the purposes of teaching and other non- commercial uses. However, the funding body(The Pharmacy Guild of Australia and the Commonwealth Department of Health and Ageing) will also retain the right to publish and use the materials and results from the study. The research contract is still under negotiation and a final copy of the contract when signed and completed will be made available to the Human Ethics Committee.

Proceed to Section 9.

SECTION 9: DESCRIPTION OF PROJECT
(refer to the National Statement on Ethical Conduct in Research Involving Humans, p. 13)

This section is obligatory

9.1 Describe the project using lay terms wherever possible, including the **aims, hypotheses, research plan** and **potential significance**. Where relevant, provide the projected number, sex, and age range of participants (including inclusion/exclusion criteria). You must satisfy the HREC that the study

is scientifically valid and conducted in accordance with the accepted ethical principles governing research involving humans.
The description must be no longer than 2 pages and must be in a font size of at least 10 points.

Completion data round up-*IN BOTH GROUPS*-

Project AIM

To develop, implement and evaluate an innovative primary care model in community pharmacy for screening, monitoring and education of people with sleep disorders and those at future risk of developing them.

Hypotheses

1. Community pharmacies can serve as effective sites for conducting a *basic screening* for patients who have or are at risk of developing a sleep disorder.
2. Additionally, pharmacists can facilitate the process of diagnoses and management of sleep disorders by conducting a *comprehensive screening* program for patients at risk of developing or having a sleep disorder, and referring, counselling and monitoring these patients.
3. A comprehensive screening can be more effective than a basic screening in detecting patients at risk of or having a sleep disorder.

Project and Research Plan

The project will be conducted in phases:

Phase 1-Development of a pharmacist sleep health education program, screening and project protocols, sleep health awareness campaign material, collaborative sleep health care kits etc. The screening tool -POTASH i.e. **P**harmacy **O**nline **T**ool for **A**ssessment of **S**leep **H**ealth, will strategically combine several validated questionnaires(Multivariate apnea prediction score, Epworth Sleepiness Score, Insomnia and International Restless Legs questionnaires)[1-4] related to various sleep disorders, as well as known risk factors, and will be an online tool to minimise paperwork and encourage completion in the pharmacy. The POTASH will be designed with the help of an expert steering committee and will aim to produce a score relating to the 'risk' of sleep disorders:

Phase 2 – **Implementation** of a sleep health awareness through participating pharmacies to raise awareness about sleep disorders in the community, recruit patients. Sample sizes and recruitment of patients and pharmacists is discussed in ***Section 3.3.***

Phase 3- Implementation of two pharmacy programs; 1) a basic screening program to determine the capacity of community pharmacies to detect undiagnosed sleep disorders in the community in all participating pharmacies AND 2) a comprehensive sleep screening and health program for those 'at risk' of developing a sleep disorder in the intervention arm. Pharmacists in both intervention and control group will administer the basic on line screening tool, i.e. POTASH. In the basic screening-(POTASH only) arm, all patients at risk of developing sleep disorders will be referred using a referral template, along with a brief report of the screening test, and a list of the patient's medications. Permission to re-contact them after 3 months will be sought.
Sequential comprehensive screening (POTASH-PLUS)arm- All patients in the intervention arm who are identified as being at risk after using the POTASH, will be provided with a nasal flow/pressure measurement device (Flow Wizard®)[5] and a Pittsburgh Sleep Diary(PSD)[6], to complete at home.
These patients will record the Flow-Wizard® readings for 3 days, and return the equipment, as well as the sleep diary to their patients (by mail/hand, as convenient). An appointment will be set by pharmacists with the patients to return to the pharmacy for a counselling/education session after the pharmacist receives the Flow-Wizard® reading results. At this session, all patients will be provided with a specialised counselling session (pre-diagnosis). This counselling session will encompass –

- *explanation of their screening results, summary of their particular risk factors (BMI -diet, exercise, poor sleep hygiene, comorbid condition, smoking, excessive alcohol intake),*
- *education about risk factor reduction(diet modification, exercise and activity,*
- *smoking cessation avenues, hypertension management, sleep habit modification, in*
- *appropriate medication use reduction, medication review etc).*
- *possible health care interventions required e.g. polysomnography, continuous*
- *positive airways pressure trial etc*
- *a sleep health information pack*
- *referral -since all these patients would have a risk of having/developing a sleep*
- *disorder based on the POTASH outcome, the pharmacist will prepare a referral to a general practitioner with a comprehensive report on the findings of the FlowWizard®) and PSD recordings and the session outcomes at the end of the pre-diagnosis session*

After 3 months, the patients will be recontacted and a closeout questionnaire about the treatments that they have received or sought in relation to their sleep disorder, or other lifestyle-behaviour modifications they have undertaken will be administered over the telephone. The close out questionnaire will also contain items on patient satisfaction, and willingness to pay for screening. Those patients who have not yet had outcomes from their referral, e.g. awaiting a polysomnograpy study, or specialist appointment will be followed up again at relevant intervals to a maximum of another two months e.g. 5 months, from baseline. Collaborating health care professionals will be contacted and a brief debrief session conducted over the

telephone to invite comments about the project. All pharmacists will be invited to participate in a debrief session about the project. 2-3 debrief sessions will be conducted for the geographic convenience of participating pharmacists.

Phase 4-Evaluation Phase

Process measures

- ♦Proportions of participating patients who self selected into the project because of the awareness campaign
- ♦Pharmacist initiated reason for recruitment of patients and rate of recruitment
- ♦Reason for refusal in patients who reject recruitment into project on invitation by pharmacists
- ♦Mean time taken to conduct the basic screening for all patients
- ♦Time taken to conduct the counselling session for patients in the comprehensive screening arm (after they have recorded FLOW-WIZARD®and Sleep Diary readings)
- ♦Patient retention in the comprehensive screening arm

Primary outcome

♦ **Proportion of patients screened who are identified as being at risk of developing a sleep disorder**

Secondary outcome

♦ ***Proportion of patients screened who are diagnosed with a sleep disorder (This evaluation will be done blinded to allocation of randomised intervention).***

Other outcomes that will be collated and where relevant, compared between the two groups

(sources of data collection may include and require liaison with GPs and specialists)

- ♦ Proportion of screened patients who take up referrals
- ♦ Proportion of patients screened who are diagnosed with *Sleep Disorders*
- ♦ Proportion of referred patients sent for further sleep specialist review
- ♦ Proportion of screened patients who initiate sleep disorder-risk factor reduction measures
- ♦ Frequency of different risk factor reduction measures undertaken by patients
- ♦ Change in pharmacists' knowledge and attitude about sleep disorders pre-post training
- ♦ Cost-analysis based on:

  1) cost of promoting the program
  2) cost of conducting the on-line screening test for sleep disorders per case of sleep disorder detected
  3) cost of conducting the Flow-Wizard/sleep diary log analysis-per case of sleep disorder detected

- ♦ Patient satisfaction with pharmacy service using open ended telephone interviews
- ♦Pharmacist and physician report on project utility, practicality and satisfaction with outcomes

Collating the above listed outcomes will help establish the effectiveness of pharmacy based screening programs for sleep disorders and help discriminate between a basic screening model and a comprehensive screening and sleep health education model.

References

1. Bastien, C. H., Vallières, A., & Morin, C. M. (2001). Validation of the Insomnia Severity Index as an outcome measure for insomnia research. Sleep Medicine, 2(4), 297-307.
2. Maislin G, Pack A, Kribbs N, Smith P, Schwartz A, Kline L, Schwab R, Dinges D(1995). A survey screen for prediction of apnea. Sleep 1995;18:158-166.
3. Allen, R. P., Picchietti, D., Hening, W. A., Trenkwalder, C., Walters, A. S., Montplaisi, J., et al. (2003). Restless legs syndrome: diagnostic criteria, special considerations, and epidemiology. A report from the restless legs syndrome diagnosis and epidemiology workshop at the National Institutes of Health. Sleep Medicine, 4(2), 101-119.
4. Johns MW. (1991) A new method for measuring daytime sleepiness: the Epworth Sleepiness Scale. Sleep; 14:540–545.
5. Monk TH, Reynolds CF, Kupfer DJ, Buysse DJ, Coble PA, Hayes AM, et al. The Pittsbugh Sleep Diary. J Sleep Res 1994;3:111---20.
6. Rogers NL, Unger G, Wong, K Hedner, JA, Grunstein R. Report on: Accuracy of a single channel nasal pressure recording Device for repeated ambulatory use in suspected sleep Apnea, Prepared for Canadian Transport: Available online at:http://www.tc.gc.ca/tdc/publication/tp14620/papers/g01.pdf, Accessed 30/04/07.

Proceed to section 10.

SECTION 10: FIELD-BASED RESEARCH (i.e., CONDUCTED OFF CAMPUS OR OUTSIDE A HEALTH SERVICE) INCLUDING RESEARCH CONDUCTED OUTSIDE AUSTRALIA

(refer to the National Statement on Ethical Conduct in Research Involving Humans, p.14, p.31-32)

This section must be completed for all applications involving EITHER field-based research OR research to be carried out in countries outside Australia (eg. in a school, a corporation, a government department an Aboriginal and Torres Strait Islander community or research in a another country).

10.1 Is your research conducted

		N	Y
**(i)**	**Outside Australia**	☒	☐
**(ii)**	**Off Campus**	☐	☑
**(iii)**	**In an Aboriginal and Torres Strait Islander Community**	☒	☐
**(iv)**	**In a School**	☒	☐
**(v)**	**In a Corporation**	☒	☐
**(vi)**	**In a Government Department**	☒	☐
**(vii)**	**In a Hospital**	☒	☐

If you answered NO to all of the above, go to Section 11

10.2 Have you obtained formal permission from relevant authorities for entry to the area to carry out research (e. g., national or local government bodies, organisations of local communities)? ☐ Y ☐ N

If you answered YES, name the relevant authorities and attach the relevant correspondence.

The research will be conducted through participating pharmacies- the owners of participating pharmacies would have consented to the research taking place on their pharmacy premises.

If you answered NO, give reasons.

10.3 If research is proposed among members of specific organisations, have you sought approval from those organisations (e. g., church groups, national associations, etc)? ☐ Y ☒ N

If you answered YES, name the relevant authorities and attach the relevant correspondence or letter of support.

NA

If you answered NO, give reasons.

The project is funded through the Pharmacy Guild of Australia- the Guild membership comprises pharmacist owners. Once the ethics approval is obtained and the research contract signed, the Guild places a list of funded projects on their website and a newsletter outlining these is sent to all community pharmacy owners. Therefore, further permission to conduct the project in community pharmacies would be a superfluous task.

10.4 Does the research involve individuals or groups of people who are not ☒ ☐

formally organised (e.g., people living in a village or town, etc)? **N** **Y**

If you answered YES, indicate the context of the research. How will you obtain access to participants? Indicate any ethical issues that you can foresee in this approach.

10.5 Will your research necessarily involve the acquisition of objects of valuable cultural property (e. g., carvings, paintings, etc)? [x] **N** [ ] **Y**

If you answered YES, give details of arrangements with owners of the property with regard to access to/acquisition of these items, where appropriate.

10.6 Will your research necessarily involve any activities that are likely to be seen by research participants and/or members of their local communities as in conflict with local practices and customs (e.g. regarding religious or ritual participation)? [x] **N** [ ] **Y**

If you answered YES, provide details.

Proceed to Section 11.

SECTION 11: RESEARCH INVOLVING BLOOD, TISSUE, ETC.
(refer to the National Statement on Ethical Conduct in Research Involving Humans, p.33, p.43-50)

This section must be completed for all research involving blood or tissue samples, or involving physical hazards.

11.1 Does this section apply to your research? [x] N [ ] Y

If NO, Go to Section 12

11.2 Will human blood or tissue be used in the research? [ ] N [ ] Y

If you answered YES, what procedures are in place to minimise the infectious and other risks to participants and researchers?

11.3 Will human embryos, fetal tissue, or placental tissue be involved? [ ] N [ ] Y

If you answered YES, provide details.

11.4 Has this blood or tissue already been collected and stored? [ ] N [ ] Y

If you answered YES, what was the original purpose of collection for the stored blood or tissue you seek to use?

11.5 Describe the proposed storage arrangements of the blood and/or tissue samples collected. Indicate how long the blood or tissue will be kept. Indicate how the samples will be disposed of upon the completion of the research.

11.6 Will genetically modified organisms or other gene modification techniques be used in the research? [ ] N [ ] Y

If you answered YES, provide details. Describe the procedures, which are in place to minimise the risks to participants and researchers.

11.7 **Will toxins, mutagens, teratogens or carcinogens be used?** ☐ N ☐ Y

If you answered YES, provide details. Describe the procedures, which are in place to minimise the risks to participants and researchers.

11.8 **Will biohazardous material be used?** ☐ N ☐ Y

If you answered YES, provide details. Describe the procedures, which are in place to minimise the risks to participants and researchers.

11.9 **Will participants or researchers be exposed to ionising radiation?** ☐ N ☐ Y

If you answered YES, provide details of the radiation exposure, including a quantitative assessment of the absorbed dose, supported either by dosimetric calculations or by other information. Describe the procedures, which are in place to minimise the risks to participants and researchers. The study should also be approved by the relevant institutional Radiiation Safety authority.

Proceed to Section 12.

SECTION 12: CLINICAL TRIALS OF DRUGS OR DEVICES
(refer to the National Statement on Ethical Conduct in Research Involving Humans, p. 35-38, and also to Therapeutic Goods Administration, http://www.tga.gov.au)

This section must be completed for all applications involving clinical trial(s).

12.1 Does this section apply to your research? ☒ N ☐ Y

If NO, Go to Section 13

12.2 (i) Is the research being conducted under the Clinical Trial Notification Scheme (CTN)? ☐ N ☐ Y

(ii) Is the research being conducted under the Clinical Trial Exemption Scheme (CTX)? ☐ N ☐ Y

(iii) **Is the research using only approved drug(s)/device(s) in accordance with Therapeutic Goods Administration Approved Product Information?** *(Note reversed order of the responses)* ☐ **Y** ☐ **N**

12.3 (a) Will this research be undertaken on behalf of (or at the request of) a pharmaceutical company, or other commercial entity, or any other sponsor? ☐ **N** ☐ **Y**

If you answered YES, provide details of the name of the sponsor (and co-sponsors if any) ?
This information should be included in the Participant Information Statement and Consent Form.

Will the sponsor(s) provide any support in money or kind?
Provide details.

(b) **If you answered YES to (a) will that entity undertake in writing to abide by either the Medicines Australia Guidelines for Injury Resulting from Participation in an Industry-Sponsored Clinical Trial (www.medicinesaustralia.com.au) or the ABPI Clinical Trial Compensation Guidelines?** ☐ **Y** ☐ **N**

If you answered NO to this question, provide details.

(c) **If you answered YES to (a), will that entity undertake in writing to indemnify the institution, the HREC(s) and the researchers ?** ***(If you answered YES, a copy of the appropriate deed or letter of indemnity should be included with the application).*** ☐ **Y** ☐ **N**

If you answered NO to this question, provide details.

(d) If you answered YES to (a), (b) or (c), does the sponsor hold a current insurance policy to cover this project? ☐ **Y** ☐ **N**
(*If you answered YES, provide a certificate of currency*).

If you answered NO to this question, provide details.

12.4 List any drugs or devices to be used, and their TGA approval status both in Australia and overseas ☐ **NA**

12.5 How many participants are projected to be enrolled into the trial at this site and in total? (Please give a single figure for each, not a range)

12.6 What is the projected duration of the trial, from first enrolment to the last protocol interaction with the last enrolled subject (in years)?

12.7 If all projected participants complete the protocol:
(a) what total payment will be received from the sponsoring company?
(Please give a single figure, not a range)

(b) what additional "in kind" support (ie free drug, equipment, etc), if any, will be provided by the sponsoring company?

For instructions on how to obtain TGA approval, please refer to **http://www.tga.gov.au**.

Proceed to the Section 13.

SECTION 13. DECLARATION OF RESEARCHERS

I/we apply for approval to conduct the research. If approval is granted, it will be undertaken in accordance with this application and other relevant laws, regulations and guidelines.

Signature of Chief Investigator or Supervisor

Name .... Signature: .... Date: ....
(print)

Signature of Associate Researcher(s) or Student(s)

Name .... Signature: .... Date: ....
(print)

Name .... Signature: .... Date: ....
(print)

Name .... Signature: .... Date: ....
(print)

Name .... Signature: .... Date: ....
(print)

Signature of appropriate senior officer NOT ASSOCIATED with the research (e.g. Head of School/ Department/Unit/Dean of Faculty or Head of Division).

After careful consideration and appropriate consultation, I have reviewed the attached HREC application, including the Participant Information Statement and Consent Form. I am satisfied that the scientific merit of this work justifies its being performed and that the information which will be obtained justifies the inconvenience and risks to participants.

Name:..
(print)

Title: ....
(print)

Position: ....
(print)

Signature: .... Date: ....

CHECKLIST FOR FULL ETHICS APPLICATION

The following documents are to be attached as indicated in the Guide to Applicants.

Check N/A if not applicable.

Have you included the **original copies (plus the number of copies required by your HREC)** of the following:

	Y	N/A
Original application	☐	
Consent form(s)	☐	☐
Participant Information Statement (s)	☐	☐
Recruitment advertisement/circular	☐	☐
Evidence of permission to conduct research in other locations	☐	☐
Evidence of approval/rejection by other HREC(s), including comments and requested alterations to the protocol	☐	☐
Copy of questionnaire(s), survey questions, interview topics to be covered etc.	☐	☐
Statement from a medical/paramedical practitioner accepting responsibility for specific procedures.	☐	☐
Risk management unit report regarding genetically-modified organisms, biohazards, Ionizing radiation, lasers or carcinogens	☐	☐
One copy of the grant application with appropriate clearance forms as requested by the Research Office **(Refer to your local requirements)**	☐	☐
Any form requiring signature by the HREC (one copy) e.g. CTN/CTX Forms	☐	☐
